# The role of cell division control protein 42 in tumor and non-tumor diseases: A systematic review

**DOI:** 10.7150/jca.65415

**Published:** 2022-01-01

**Authors:** Junjie Fu, Bo Liu, Hao Zhang, Fangmei Fu, Xiaohui Yang, Linlin Fan, Minying Zheng, Shiwu Zhang

**Affiliations:** 1 Graduate School, Tianjin Medical University, Tianjin, 300070, P.R. China.; 2 Medical Affairs Office, Tianjin Union Medical Center, Tianjin, P.R. China.; 3 Graduate School, Tianjin University of Traditional Chinese Medicine, Tianjin, P.R. China.; 4 Nankai University School of Medicine, Nankai University, Tianjin, P.R.China.; 5 Department of Pathology, Tianjin Union Medical Center, Tianjin, P.R. China.

**Keywords:** cell division control protein 42, effector proteins, malignant tumors, benign diseases

## Abstract

Rho-GTPases control a variety of cellular functions mainly by regulating microtubule and actin dynamics, affecting the cytoskeleton, and are important regulators of the structural plasticity of dendrites and spines. Members of the Rho-GTPase family include Ras-related C3 botulinum toxin substrate 1 (Rac1), RhoA (Ras homologous), and cell division control protein 42 (Cdc42). Cdc42 is involved in the regulation of a variety of tumor and non-tumor diseases through a cascade of multiple signaling pathways. Active Cdc42 can regulate intercellular adhesion, cytoskeleton formation, and cell cycle, thus affecting cell proliferation, transformation, and dynamic balance as well as migration and invasion of tumor cells by regulating the expression of effector proteins. Here we discuss the role of Cdc42 in promoting metastasis, invasion, epithelial-mesenchymal transformation and angiogenesis in malignant tumors. The significant role of Cdc42 in non-tumor diseases is also discussed. Since Cdc42 plays a central role in the development of various diseases, small molecule inhibitors targeting Cdc42 have important clinical significance in the prevention and treatment of these diseases.

## Background

Rho-GTPases are a family of small GTP-binding proteins in the Ras superfamily, and their relative molecular weight is approximately 20-25 KD. Rho is found in all eukaryotes from plants to humans. Twenty members of the Rho family are divided into classic and non-classic groups. The peptide chain length of a member of the classic family is approximately 3-4 times that of a member of the non-classic family. The classic Rho-GTPase group includes RhoA (Ras homologous), Ras-related C3 botulinum toxin substrate 1 (Rac1), and cell division control protein 42 (Cdc42), which are modulated by the effects of Rho-specific guanine nucleotide exchange factor (GEF) and GTPase activating proteins (GAPs) and guanosine nucleotide dissociation inhibitors (GDIs). Rho-GEF regulates the exchange of GTP with GDP, thereby activating Rho-GTPases. Simultaneously, GAPs accelerates GTP hydrolysis and restores these proteins to an inactive state. Post-translational modification and the GTP/GDP cycle together regulate the biological activity of Rho-GTPases 1. Cdc42 is one of the most important members of the Rho-GTP family and it is found first in fission yeast. It plays a crucial role in regulating the cell cycle, controlling gene transcription, regulating the cytoskeleton, cell movement, and polarization. Cdc42 activates the c-Jun NH2-terminal kinase (JNK) and mitogen-activated protein kinase (P38/MAPK) pathways to control gene transcription. In addition, Cdc42 can activate neural Wiskott-Aldrich syndrome protein (N-WASP) and form a complex with actin monomer binding proteins, G-actin and actin-related protein 2/3 (Arp2/3), which promotes the aggregation of F-actin, and produces filopodia and induces cytoskeleton changes. P21-activated kinases (PAKs) are Cdc42 effector proteins, and they are cytosolic serine/threonine protein kinases, which can bind to a polarity scaffold protein or interact with Cdc42-directed GEF 2-7. Here, we systematically reviewed the structure, functions, and small molecule inhibitors of Cdc42 in malignant tumor and non-tumor diseases, providing specific targets for the treatment of Cdc42-related diseases.

## Structure and function of Cdc42

Cdc42 is a member of the Rho family with a molecular weight of 21 KD and is first discovered in yeast, *Saccharomyces cerevisiae* 8. There are six kinds of Rho-GTPases in yeast, which are critical for maintaining cell polarity on the cell membrane. Cdc42 has two different isoforms, Cdc42a (also known as Cdc42p and Cdc42Hs) and Cdc42b (also known as brain-derived Cdc42p or G25K GTP-binding protein). Cdc42a is widely expressed human tissues whereas Cdc42b is only expressed in the brain. The human Cdc42 gene is located on chromosome 1p36.1. The Cdc42 protein contains 191 amino acids and is mainly located in the mitotic spindle, cytokinesis central spindle, and intermediates.

Cdc42 is particularly important in yeast germination 9, 10 and polarization during growth, by promoting cytoskeleton remodeling and vesicle exocytosis, which are required for normal cell differentiation and movement 11, 12. When Cdc42 function is lost, cells become round and budding growth is inhibited 12, 13. In yeast cells, polarization of the cytoskeleton and the membrane transport system requires the isotropic and polarized distribution of Cdc42. Other studies have demonstrated that cytoskeleton and membrane transport systems have a negative feedback effect on Cdc42 distribution 14-16. When yeast cells sprout, Cdc42 is first localized in the cortical region of the cell membrane and forms a complex with septin family members (Cdc3, Cdc10, Cdc11, and Cdc12). Then, septin family members are assigned to the specific growth site of the budding to participate in the generation of daughter cells 17, 18.

Cdc42 regulates microtubule cytoskeleton through microtubule reorganization, including asymmetric localization of the spindle. In addition, Cdc42 facilitates the development of the pancreas, blood cells, eyes, and skin 19. Cdc42 is activated when it binds to GTP, but inactivated when it binds to GDP, and the activity of Cdc42 is regulated by GEFs, GAPs and GDIs 20. The GAPs are designed to inactivate Cdc42 by hydrolyzing GTP into GDP. Most of the GAPs have non-specific effects on different small GTPases. GAPs includes Rga1, Rga2, and Bem3 21. Rga1 and Rga2 mainly regulate the interaction between Cdc42 and Ste20. Bem3 mediates the interaction between Cdc42 and Cla4 during cell division 21. GDIs can block Cdc42 binding with GDP in cytoplasm and inhibit the activity of Cdc42. GDIs consist of an immunoglobulin-like C-terminal structure, and a flexible N-terminal domain that inhibits the exchange of GDP with GTP 22.

**Figure 1:**
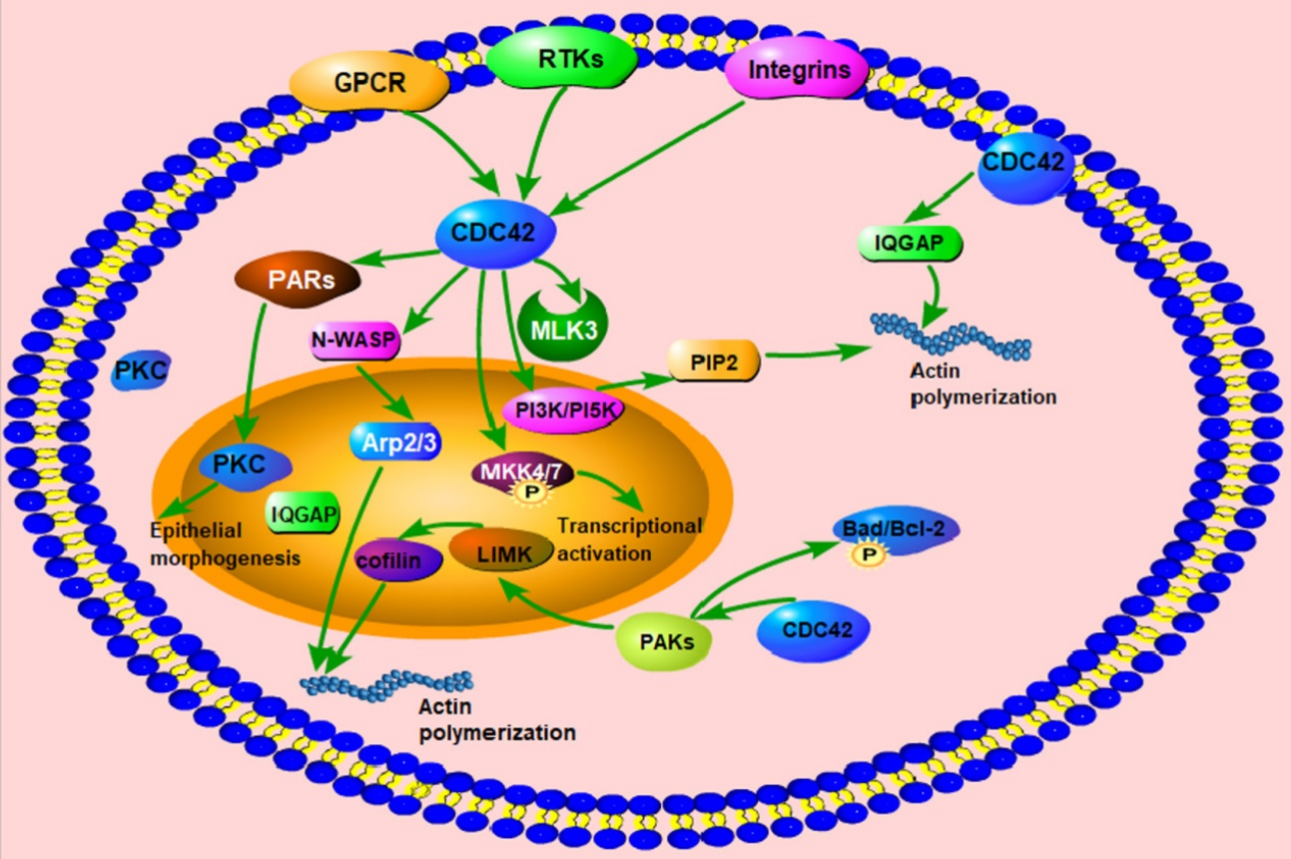
The function and regulatory proteins of Cdc42. Cdc42 can activate downstream signaling molecules, such as PI3K/PI5K, N-WASP, WASP, PAK1/2 and IQGAP, to regulate the structure and function of actin, including actin polymerization, actin assembly, actin cytoskeleton regulation, actin stabilization, etc. Cdc42 activates MLK3 and phosphorylates MKK4/7 to participate in the transcriptional activation process. Cdc42 activates the PARs complex and PKC to promote epithelial morphogenesis. Cdc42 participates in the regulation of actin dynamics, and cell survival through phosphorylation of Bad/Bcl-2.

Active Cdc42 can regulate the expression of downstream effectors, and enhance cell proliferation, polarity, adhesion, and migration, as well as dynamic changes of the cytoskeleton 23. Cdc42 accumulates in the cellular cortical cap to maintain cell polarity and regulates the polymerization and dissociation of cytoskeletal actin 24, 25. Cdc42 can be activated when bradykinin binds to the corresponding receptors on the cell membrane. Activated Cdc42 can regulate the assembly of the plaque complex on the cell membrane and enhance the secretion of mastocytes as well as promote the transport of vesicles from the Golgi to the plasma membrane. Mutations rarely occur in Cdc42 26. When it mutates, it can combine with the γ subunit of the coatomer complex to regulate the material transport from the rough endoplasmic reticulum to the Golgi apparatus. Meanwhile, the γ subunit plays a continuous activating role in Cdc42-induced malignant transformation 27 (Fig. 1).

## Cdc42-related proteins and signaling pathways

### Cdc42 regulates the recombination of cytoskeletal proteins via P21-activated kinases (PAKs)

PAKs are downstream proteins of Cdc42 and are known for their roles in the cytoskeleton and are critical in the dynamic recombination of F-actin and tubulin. The PAKs family contains six proteins: PAK-1(α-PAK), PAK-2 (γ-PAK), PAK-3 (β-PAK) (group Ⅰ), PAK-4, PAK-5, and PAK-6 (group Ⅱ). Group Ⅰ PAKs have an autoinhibitory domain and can interact with the kinase domain in a cis-autoinhibitory interaction. Binding of GTPase to PAKs disrupts this autoinhibition, leading to dimerization and trans-autophosphorylation of the two PAK monomers 28. Group Ⅱ PAKs have constitutive activation loop autophosphorylation, which is not affected by the binding of Rho GTPase 29. Group II PAKs are activated by the dissociation of a proline-rich psudosubstrate region from the kinase domain 30. PAK-1 is important in the formation of lamellipodia and it is an interface between adhesion junctional complex (AJC) destabilization and increases motility 31. Upon activation of Cdc42, PAK-1 phosphorylates LIMK and further inactivates cofilin. During the G1 phase of the cell cycle, PAK-1 activates extracellular signal-regulated kinase (ERK) and nuclear factor ƙB (NF-ƙB) to increase cyclin D1 transcription. Late in the cell cycle, PAK-1 is activated in the centrosome, resulting in phosphorylation of Polo-like kinase 1 and Aurora A kinases, which regulates the mitotic process 32. PAK-2 promotes cell survival by phosphorylating Bad and Bcl-2 33. On the other hand, apoptotic stimuli such as DNA damage can lead to caspase-mediated cleavage of PAK-2, resulting in the generation of p34 fragments. PAK-2-p34 activates JNK and inactivates MNK, leading to inactivation of the mitochondria-dependent death pathway 34. Multiple human cancers are associated with overexpression of PAK-1, such as non-small-cell lung cancer 35, breast cancer, prostate cancer 36, pancreatic cancer, gastric cancer 37, colorectal cancer 38, and oral cancer 39. Similarly, PAK-2 is highly expressed in gastric and ovarian cancers. In humans, deleterious mutations in the PAK-3 gene have been confirmed to be associated with X-linked non-syndromic intellectual disability through the JNK and P38 pathways 40. Chung, E. Y. et al reported that inhibition of PAK2 could reduce CADM1-mediated stromal interactions and promote survival of adult T-Cell leukemia/lymphoma cells 41. Moreover, PAK-4 gene amplification has been found in colorectal and pancreatic cancers via the phosphatidylinositol 3-kinase (PI3K) mutation 42, 43. PAK-5 is dysregulated in ovarian cancer through the activation of the PI3K/AKT pathway, in cervical cancer via special AT-rich binding protein-1, and in glioblastoma via inhibition of early growth response protein 1 to upregulate matrix metalloproteinase 2 44. Finally, PAK-6 promotes cervical cancer progression through activation of the Wnt/β-catenin signaling pathway 45.

### Cdc42 binds activated Cdc42 kinase (ACK) to promote metastasis

ACK is a non-receptor tyrosine kinase and functions downstream of growth factor receptors and has been implicated in survival, neuronal signaling, and androgen receptor activation through identification of its phosphorylation targets. The conformation of ACK binding to Cdc42 is significantly different from that of other CRIB effector proteins, all of which form a short segment of intermolecular β-sheet with Cdc42. Leu449, Ser-450 and Ala-451 residues in ACK are the key factors for binding to Cdc42 46. They interact with Cdc42 via helix α5 and the C-terminus of the protein. Leu174 is the specific determinant of ACK binding to Cdc42 47. In the N-terminal of ACK, Thr-35 can interact with both the γ-phosphate of GTP and Mg^2+^ to form a complex, which binds to Cdc42 in an initial and transient manner. The C-terminal region of the ACK contributes to the overall binding affinity through several scattered residues 46. ACK1 is a member of the ACK family which consists of the N-terminal sterile topic domain, amino N-terminal Src-homology 3 (SH3) domain, kinase domain, and Cdc42/Rac interactive binding (CRIB), a proline-rich domain. Cdc42 can activate ACK1 by directly and specifically binding to it 48, which enables cells to bypass the blockade to major survival pathways to promote cancer progression and resistance to standard cancer treatments. In triple-negative breast cancer, ACK1 is highly expressed and triggers the activation of PI3K, which aggregates in the cell membrane and activates protein kinase B, enhancing the progression of breast cancer 49. In addition, low expression of ACK1 can reduce the ubiquitination level of P53, thereby inducing G2/M phase arrest and apoptosis of gastric cancer cells, and preventing the occurrence of gastric cancer cells 50.

### Cdc42 enhances cell proliferation and migration by activating lipid kinases

PI3K and PI5K are the core effector proteins of Cdc42 /Rac. Cdc42 /Rac and PI3K are mainly present in the mammalian system. PI5K and PI3K can produce PIP2 and act as a second messenger to regulate the dynamic balance of the cytoskeleton**.** PI3K I has three homologous isoforms: PI3KCA, PI3KCB, and PI3KCD. PI3KCA mutations have been found in colon and non-small-cell lung cancer 51, 52. However, among these three kinds of homologous forms, PIK3CB is more important in cancer than the other two. PI3KCB can be activated by Cdc42 and Rac1, and inactivation of PIK3CB can significantly suppress tumor proliferation, metastasis, and invasion in in PTEN-deficient cancers 53.

Isoleucine-glutamine-motif containing GTPase-activating proteins (IQGAPs) promotes cell metastasis and invasion by binding to the activated Cdc42-GTP. IQGAPs are downstream effectors of Cdc42 and consist of two key regions, including a calmodulin binding IQ-motif and a GTPase activating protein related domain. Upregulated IQGAPs bind to the activated Cdc42-GTP 54 and promote cell metastasis and invasion 55, and the formation of the cytokinetic actin ring. IQGAPs can be divided in three groups: IQGAP 1, 2, and 3. IQGAP1 can intensify the regulatory ability of Cdc42p in the formation of the actin cytoskeleton 56, 57 and promote the occurrence and metastasis of carcinoma. IQGAP1 is associated with adhesion junctions and it can reduce E-cadherin mediated cell adhesion involved in tumor progression 58-62. Studies have shown that IQGAP1 is highly expressed in cancer and is closely related to metastasis potential. Many studies have explored the Wnt/E-cadherin/β-catenin pathway downstream of IQGAP1 as a potential target for gastric cancer 63-65, and it has been reported that IQGAP1 gene knockout leads to gastric dysplasia in mice 66. IQGAP1 binds to epidermal growth factor receptor (EGFR) and acts on epidermic growth factor (EGF) to activate AKT, weakening the effects of mammalian target of rapamycin complex, glycogen synthase kinaseM, and ERK1/2, inhibiting cell secretion, and promoting cell transformation 55, 67. There are only a few studies on IQGAP2 and IQGAP3. IQGAP2 is 62% identical to IQGAP1 and has been reported to interact with both GDP- and GTP-bound forms, and may be a suppressor of cancer 57, 68. IQGAP3 has rarely been reported and its specific role is unclear, although it has been suggested to be related to cell proliferation through the Ras/ERK signaling cascade 69.

### Formins can regulate actin dynamics by binding to activated Cdc42

Formins are defined by a unique and highly conserved C-terminal formin homology 2 (FH2) domain and an N-terminal proline-rich formin homology 1 (FH1) domain 70. FH1 has approximately 100 amino-acid and FH2 has approximately 130 amino acids. A highly structured homodimer of FH2 nucleates actin filaments. Formins can bind to activated Cdc42 via their FH1 domain. Furthermore, formins have different classifications and play different functions in different species. In budding yeast, the formin Bni1p associates with Cdc42p to regulate actin dynamics 71. In *Drosophila melanogaster*, the gene encoding the formin protein Diaphanous is involved in cytokinesis 72. In *Caenorhabditis elegans*, loss of the formin protein Cyk-1 function causes polar-body extrusion in embryonic mitosis 73. The expression levels of formin-like 2 (FMNL2) are elevated in metastatic colorectal cancer cells 74 and decreased in hepatocellular carcinoma cells 75. The reduction in the expression level of formin-homology-2-domain-containing protein through miRNA regulation inhibits the invasion of breast cancer 76. When mutations occur in exon 4, which encodes for inverted FMNL2, the occurrence rate of autosomal dominant focal and segmental glomerulosclerosis is increased 77.

### Cdc42 activates neural Wiskott-Aldrich syndrome protein (N-WASP) to promote actin polymerization

N-WASP is composed of GTPase-binding domain, CRIB and a VCA domain. Cdc42 interacts with and activates N-WASP. N-WASP then binds and activates the actin-associated protein 2/3 (Arp2/3) complex, leading to actin polymerization 78. Cdc42-dependent actin assembly 1 (Toca-1) is involved in this process. Toca-1, Cdc42 and N-WASP form trimer complex, which promotes the assembly of Toca-1/N-WASP complex 79. N-WASP also can be activated by binding to phosphatidylinositol bisphosphate-PIP2 through a pleckstrin-homology lipid-interaction domain. Hou et al. reported that N-WASP had the opposite function on tumor progression. Overexpression of N-WASP enhanced the invasion and migration of cervical cancer cells by regulating the P38/MAPKs signaling pathway 80. In addition, overexpression of N-WASP correlates with the infiltration depth, lymph node metastasis, and pathological staging in esophageal squamous cell carcinoma (ESCC) 81. However, Martin, T. A. reported that the expression of N-WASP was lower in breast cancer tissues compared with that in normal mammary tissues, and the invasiveness and migration of MDA-MB-231 cells were inhibited after transfected with N-WASP 82.

### Cdc42 regulates cell polarity via binding protease-activated receptors (PARs)

The PAR protein family contains from PAR1 to PAR6. PAR3, PAR6 and atypical protein kinase C (aPKC) form a ternary complex named PARs complex that localizes asymmetrically in polarized cells, while PAR1 localizes to complementary domains. PAR1 phosphorylates PAR3 to block PAR3 binding to aPKC and inhibits cortical association of PAR3/PAR6/aPKC in *Drosophila* 83. aPKC can phosphorylate PAR1 kinases to regulate localization and activity 84. PAR5 promotes the interactions by binding phosphorylated forms of both PAR1 and PAR3. PAR4 is necessary for normal PAR asymmetries, and can drive polarization of single kidney intestinal epithelial cells after activated 85. Cdc42 is activator of PAR3/PAR6/aPKC complex. Cdc42 controls formation of the PAR3/PAR6/aPKC complex, which plays a crucial part in *Drosophila* neuroectoderm and neuroblast development and mammalian epithelial cell apical-basal polarity and axon-dendrite polarity of neuron 86. In addition, Cdc42 mediates the formation of PAR6/PKCζ complex, which regulates phosphorylation of GSK-3β to regulate the cell polarity.

### Cdc42 mediates cell proliferation, transformation, and apoptosis by activating mixed lineage kinase 3 (MLK3)

MLK3 is a member of the mixed spectrum kinase family (MAPK). The MAPK family is composed of an amino N-terminal SH3 domain, a catalytic domain, leucine/isoleucine zipper motifs, and a CRIB motif. Activated Cdc42 interacts with MLK3 through CRIB motif and promotes the catalytic activity of MLK3. In a Cdc42-mediated MLK3 activation model, Cdc42 binds to the CRIB domain of MLK3 and blocks the association between the N-terminus of MLK3 and proline 495, thereby reducing the autoinhibition of MLK3 and forming an open structure that promotes autophosphorylation 87. Activated MLK3 activates JNK by directing phosphorylation and activation of mitogen-activated protein kinase kinases (MKK) 4 and MKK7. Meanwhile, MLK3 is involved in cell proliferation, transformation, and apoptosis via mitogen-dependent activation of ERK 88, and promotes the invasion and metastasis in non-small cell lung cancer, breast cancer, melanoma, and ovarian cancer cells 89-91.

### MAPK is involved in the regulation of Cdc42 activity

MAPK signaling pathway consists of MAPKKK, MAPKK and MAPK, and they undergo phosphorylation in turn in response to internal and external stimuli. When these three kinases are activated, phosphorylated MAPK (ERK) will migrate to the nucleus and phosphorylate nuclear transcription factors to sequentially phosphorylate of specific transcription factors and then initiate transcription process 92. There is a functional interaction between Cdc42 GTPase and MAPK Sty1. The function of MAPK Sty1 promoting Cdc42 dispersal is independent of stress-induced gene expression via Atf1 or plo1-mediated regulation of cell polarity. The amino acid sequence of Cdc42 has a single consensus site for MAPK phosphorylation. Mutavchiev et al found that MAPK may negatively regulate the coupling of the Cdc42 module to cell-polarity landmarks 93.

### Cdc42 and Eph-Ephrin signaling pathway

EphA regulates actin cytoskeletal dynamics through Rho family GTPases. The Rho-GEF ephexin, associates with EphA receptors can activate Cdc42. After EphA binding, ephexin becomes phosphorylated and increased Rho activation, which leads to a destabilization of the actin cytoskeleton. A number of Rho family GAPs and GEFs are activated by EphA forward signaling. The GAP α2-chimaerin is the major GAP downstream of EphA4 in midline guidance processes. α2-chimaerin binds to the activated EphA4 receptor 94. Ephexin is a GEF that when bound to EphAs in the absence of ephrin-A binding causes activation of Rho, Cdc42, and Rac1 95. When EphAs are bound to ephrin-As, ephexin changes its activity, which increases the Rho activation and decreases the activation of Cdc42 and Rac1.

## Cdc42 and malignant tumors

**Figure 2:**
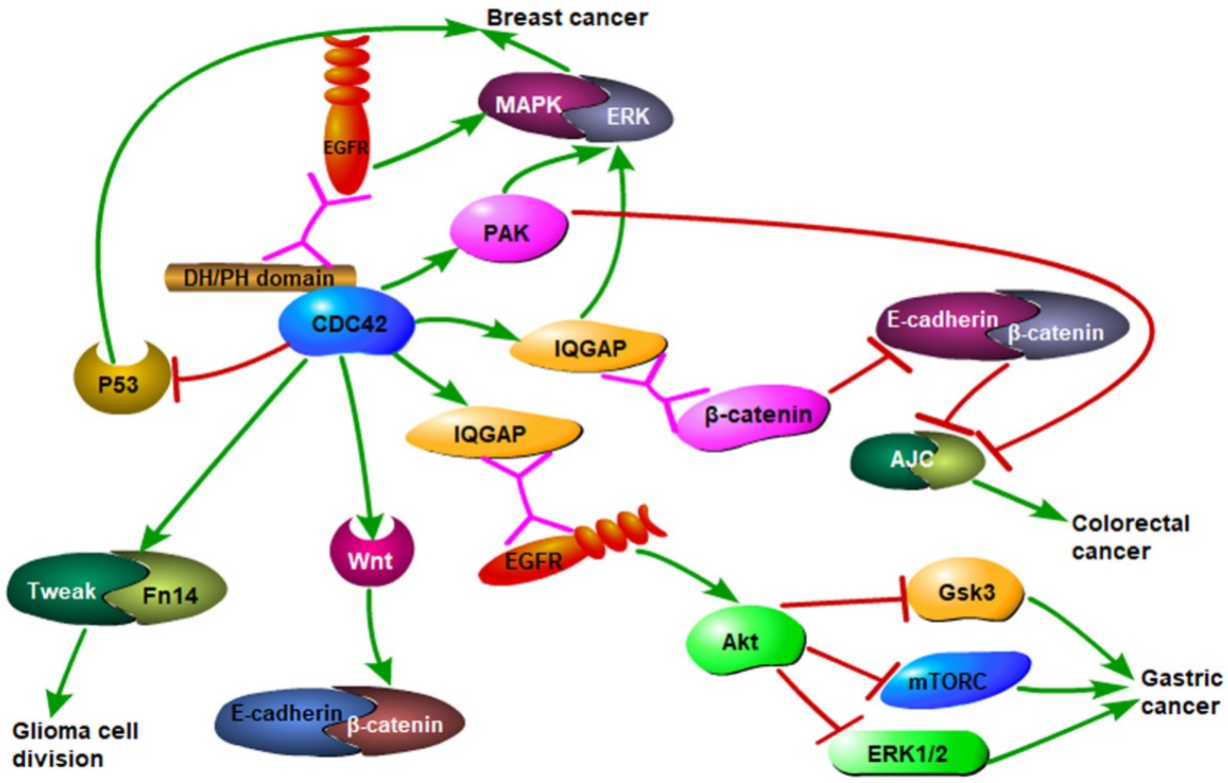
Cdc42 mediates the occurrence of multiple malignant tumors through different pathways. Cdc42 binding to EGFR receptor activates MAPK/ERK signaling pathway, leading to the development of breast cancer. It also activates downstream effector molecules PAK and IQGAP, which are related with breast cancer occurrence. Cdc42 activates the WNT signaling pathway and AKT, and then inhibits GSK3, mTORC, and ERK1/2, leading to the development of gastric cancer. Cdc42 activates Tweak/Fn14 complex and leads to glioma formation. Cdc42 also inhibits the formation of adhesive junction complexes (AJC) giving rise to the development of colorectal cancer.

Although Cdc42 is first found in yeast, where it plays an important role, it is equally important in the occurrence and development of various human malignant tumors. In most human normal tissue cells, Cdc42 expression is low/medium both at the mRNA and protein level. However, in tumor cells, the high expression of activated Cdc42, abnormal ubiquitin ligase, and EGFR degradation inhibition result in malignant tumor progression 96. Cdc42 activates downstream actin-related proteins and induces fasciculation of F-actin in the filamentous pseudopodia of the cell, adheres to the extracellular matrix, and promotes cell movement. In ovarian cancer, breast cancer, bladder cancer, and prostate cancer, the downstream PAKs of Cdc42 are highly expressed 97, 98. Additionally, the growth of tumor cells usually consumes a lot of glucose and glutamine under aerobic conditions, which is supplemented by the tricarboxylic acid cycle, which enables sustained biosynthesis of lipids, proteins, and nucleic acids and promotes cell proliferation. Mutation of Cdc42 can increase the activity of glutamine in the mitochondria, which can cause continuous metabolism of glutamine and change the metabolic mode of tumor cells, leading to malignant development 99 (Fig. 2). Hadad, S.et al reported that metformin reduced the incidence of breast cancer and enhanced response to neoadjuvant chemotherapy in diabetic women by upregulating TGFB and MEKK and downregulating Cdc42 100.

### Cdc42 promotes proliferation of cancer cells

Cdc42 is generally upregulated and involved in the activation of cell signaling pathways, including PAK and N-WASP, which are closely related to the malignant proliferation of tumor cells 101. Cdc42 can enhance tumor cell proliferation by regulating the expression of EGFR in gastric cancer 102 103. Meanwhile, the proliferation of breast cancer cells requires the nutrition supply of new blood vessels and the expression of angiogenic factors (EGF, vascular endothelial growth factor (VEGF), interleukin (IL-6, and IL-8) which are regulated by Cdc42 104. In colorectal cancer, Cdc42 is involved in cancer cell proliferation through the PAK-mediated tight junction and AJC. The functional tight junction and AJC inhibit cell proliferation and lead to the destruction of intercellular adhesion, indicating that Rho-GTPases can directly participate in regulation of proliferation. The deficiency of Cdc42 can lead to an imbalance in the proliferation and apoptosis of breast epithelial cells by inactivating cyclin D1, which hinders the G1/S transition. Second, the lack of Cdc42 prevents the formation of a complex with PAR and PKC, which results in the failure of apical polarity formation in mammary epithelial cells 105 and changes in spindle orientation. Additionally, in breast cancer and colorectal cancer, Cdc42 regulates anti-apoptotic signals through the anti-cancer gene p53 106, anti-apoptotic Bcl-2 family members, proto-oncogene C-myc*,* and fatty acid synthase, supporting breast cancer cells continuous growth 107.

### Cdc42 promotes epithelial-mesenchymal transition (EMT)

EMT is a major driver of cancer cells invasion and metastasis. EMT allows cancer cells to metastasize and break the adhesion between cells. EMT plays an important role in physiological and pathological processes, including the promotion of wound healing, tissue fibrosis, and cancer progression. EMT and the formation of new blood vessels are the basis of tumor metastasis. EMT is a complex cellular process characterized by the loss of E-cadherin and adhesion complex, and is regulated by EMT-induced transcription factors (such as Snail, Slug, and Twist) 108, 109. Besides E-cadherin, β-catenin, α-catenin, and p120-catenin are involved in intercellular adhesion. P120-catenin dissociates from adhesion junctions and localizes in the cytoplasm where it can interact with Cdc42, leading to enhanced metastasis and invasion capabilities of cancer cells. Moreover, p120-catenin activates Rac1 and Cdc42 indirectly by activating the guanine nucleotide exchange factor Vav2. A study by Bisson N showed that inhibition of Rhov, Rac1, and PAK could inhibit EMT transcription factors in *Xenopus laevis*, indicating that Rhov, Rac1, Cdc42, and their downstream effectors PAK-1 was involved in EMT 110. Cdc42 also participates in the EMT process through IQGAP1. When Cdc42 is inactivated, IQGAP1 binds to β-catenin, resulting in the loss of α-catenin-linked actin filaments and reduces adhesion between cells. Active Cdc42 binds to IQGAP1, resulting in reduced Cdc42/β-catenin interaction 111. Interferon regulatory factor 4 binding protein (IBP) can mediate the activation of RAC1, RhoA, and Cdc42, to regulate actin cytoskeletal rearrangement and produce MMPs. IBP can mediate the phenotype of EMT by down-regulating the expression of E-cadherin and keratin and up-regulating the expression of N-cadherin to increase the invasion and metastasis ability of breast cancer cells 112.

### Other proteins involved in Cdc42 regulating the invasion and metastasis of cancer cells

During colorectal cancer progression, there are two patterns of metastasis including mesenchymal metastasis and amoeba metastasis. The switching of tumor cell metastasis patterns depends on the response to Rho-GTPases 113. Cdc42 induces actin polymerization at the front edge of the lamellar lipid membrane to promote the metastasis of colorectal cancer cells. Tight junctions, adhesion junctions, and AJC contribute to form the connections of gastrointestinal epithelial cells. Among the effector proteins of Rho-GTPases, Rho-associated protein kinases are involved in the decomposition of AJC and regulate cell polarity and metastasis of colorectal cancer cells. Rho-GTPases can affect adenomatous polyposis coli (APC) and APC activates the specific GEF for Cdc42 114. Minard, M.E, et al have shown that triggering Tiam1, a GEF of both Rac and Cdc42, can induce the downregulation of E-cadherin and increase the expression of vimentin to increase the migration ability of colorectal cancer cell 115. In glioma, Slit homolog protein/roundabout homolog 1 (Slit-Robo1) can reduce the activity of Cdc42 to inhibit the invasion of cancer cells 116. TNF-like weak inducers of apoptosis (TWEAK) and fibroblast growth factor inducible-14 (Fn14) can activate Ect2-GEF and Cdc42 117 to regulate the metastasis and invasion of glioma 117. The receptor proteins tyrosine phosphatase and dynamin 2 also participate in the metastasis of glioma cells and activate Rac1 and Cdc42. Zhang, et al. reported that the loss of Cdc42-specific Rho GTPase-activating protein 21 caused morphological changes in glioma cells, and increased Cdc42 activity and cell migration ability. The downstream of the Slit2/Robo1 signaling pathway is Slit-Robo1 GTPase-activating protein (srGAPs) including srGAP1, srGAP2, and srGAP3. SrGAP1 interacts with the intracellular domain (CC3 motif) of Robo1 during neuronal migration, leading to the repelling activity of Slit and the inhibition of Cdc42 118. When Slit2 is overexpressed, the GTPase activity of Cdc42 decreases, which inhibits Cdc42 expression 118.

### Cdc42 promoted the migration and invasion of daughter cells derived from PGCCs

We previously reported that polyploid giant cell cancer cells (PGCCs) could be induced and purified by cobalt chloride. PGCCs were found to be in a dynamic equilibrium with diploid cancer cells and could be formed through endoreduplication or cell fusion 119. They reverted to regular cancer cells via asymmetric cell divisions, including the splitting, budding, or burst-like mechanisms commonly used in the replication of low-level eukaryotes, plants, and viruses 85. PGCCs produce daughter cells via asymmetric cell division and the daughter cells have strong ability of migration, invasion and proliferation 119. In an unpublished paper, we confirmed that Cdc42 involved in process of PGCCs producing daughter cells. Daughter cells derived from PGCCs have strong abilities of proliferation, migration and invasion 120. The results of western blot showed that the expression of Cdc42 and the downstream protein PAK1 in PGCCs and their daughter cells treated by cobalt chloride was significantly higher than that of the LoVo and Hct116 control cells.

### Cdc42 facilitates tumor angiogenesis

Hypoxic inducible factor (HIF), cyclin D1, IL-6, IL-8, fibroblast growth factor (FGF), and integrins, are associated with the process of angiogenesis. HIF induces gene transcription of vascular endothelial growth factor, nitric oxide synthase, platelet-derived growth factor-β, and angiopoietin 2 during EMT 121. Hypoxia increased the expression of Cdc42, Rac1, and RhoA in human cancer cells and microvascular endothelial cells 122. During angiogenesis, the proliferation of endothelial cell requires full factor signal transduction and integrin signaling to activate cyclin D1, which is a cell cycle-related protein. At the same time, activation of cyclin D1 requires ERK activation. RhoA, Rac1, and Cdc42 are necessary to maintain the activity of ERK 123. IL-6 and IL-8 can promote tumor growth and angiogenesis 124, 125, 126, 127, which can be induced by hypoxia and upregulated VEGF mRNA expression 128. Increased Cdc42 activity increases the activation of NF-ƙB, resulting in increased accumulation of IL-6 mRNA and IL-8 mRNA and protein 129, 130. In addition, FGF is an important angiogenic protein, and Cdc42 increases FGF1 expression by binding to the Ets site of the FGF1D promoter 131, stimulating the FGF1 gene promoter region. Integrins and the Rho signal transduction pathway are interrelated at many levels and together promote the development of angiogenesis.

## Cdc42 and human benign diseases

**Figure 3:**
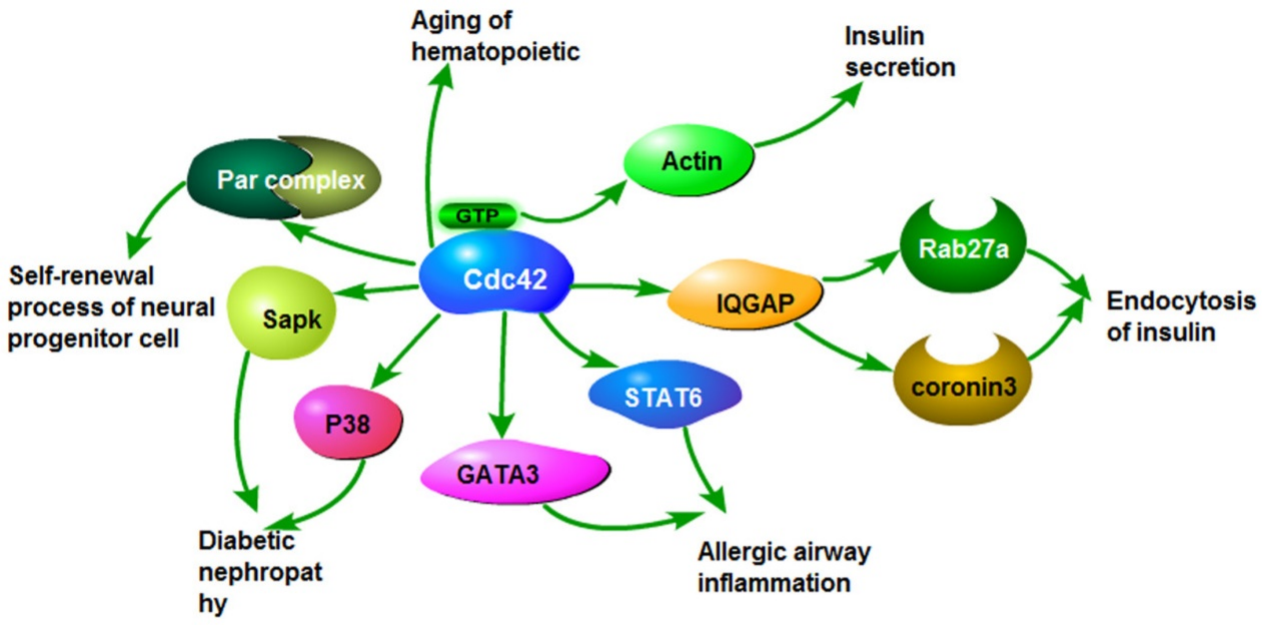
The roles of Cdc42 in non-tumor diseases. Cdc42 activates stress-activated protein kinase and P38, which causes diabetic nephropathy. Cdc42 activates GATA3 and STAT6, leading to airway inflammation. Cdc42 induces insulin endocytosis by activating Rab27a and coronavirus 3. Cdc42 binding GTP activates actin and causes insulin secretion. Cdc42 causes aging of hematopoietic stem cells. Cdc42 activates the Par complex, involved in the self-renewal of stem/progenitor cells.

Cdc42 participates in the regulation of a variety of biological processes. The increased expression of Cdc42 can promote the proliferation, invasion, and migration of cancer cells, and accelerate malignant progression of cancer. Overexpression or loss of Cdc42 also contributes to the development of some benign diseases through the same or similar signaling pathways as malignant tumors, such as insulin secretion, insulin resistance, airway inflammation and neurodegenerative diseases (Fig.3).

### Cdc42 is involved in diabetes via regulating PAKs and IQGAP

The constitutive activity of Cdc42 can interfere with the differentiation of β cells, inducing the rise of blood glucose sometimes leading to hyperglycemia. In normal human cells, Cdc42 regulates the formation of normal islet morphology associated with β cell function and proliferation 132, which is mainly accomplished by regulating the downstream signaling molecules PAK1 and cyclin D1. Cdc42 regulates IQGAP1, activates Rab27a and coronin-3, and adjusts the redistribution of Rab27a and coronin3 to regulate the function of cell membrane endocytosis 133, 134. The decrease in Cdc42 can inhibit the activity of PAK1 and increase the risk of insulin resistance 135, 136. The decrease in Cdc42 activity and G protein-coupled receptor kinase 2 (GRK2) activity leads to the downregulation of insulin-stimulated glucose transporter 4 translocation, which ultimately leads to the inhibition of insulin-stimulated glucose transport and insulin desensitization 137. Podocytes affect cytoskeletal actin dynamics and Cdc42 can regulate the expression of actin. When Cdc42 is dysfunctional, the stability and rearrangement of the cytoskeleton can lead to adhesion impairment in podocytes. The expression of high-sensitivity Cdc42 activates SAPK/P38 pathways to promote mesangial cell hypertrophy and migration 138. Furthermore, loss of Cdc42 is also an important cause of renal tubulointerstitial fibrosis in congenital nephrotic syndrome and glomerular sclerosis 139.

### Cdc42 dysregulates transducer and activator of transcription 6 (STAT6) and GATA-binding protein 3 (GATA3) to lead airway inflammation

Signal transducer and activator of transcription 6 (STAT6) can be activated by IL-4 and IL-13. GATA3 has been demonstrated to play important roles in innate lymphoid cells and to regulate T cell development, proliferation, and maintenance, in addition to controlling Th2 differentiation 140, 141. The lack of Cdc42 in T cells can significantly decrease the activation and expression of STAT6 and GATA3. These two transcription factors are crucial for the secretion of IL-4 and the differentiation of Th2 cells. When Cdc42 is deficient, the homeostasis of peripheral T cells is disrupted, and the activation of CD4^+^Foxp3^+^ reduces regulatory T cells, inhibits the development of thymus cells, and suppresses the differentiation of Th1/Th17 cells 142, 143. Cdc42 regulates both cell activation and clonal expansion in CD4^+^ T cells. In contrast, Cdc42 regulates clonal expansion but not activation in CD8^+^ cells 144.

### Cdc42 dysregulates the neurite formation and results in neurodegenerative diseases

Endo, M., J.E. et al. confirmed that Cdc42 could stimulate the activity of mTOR complex 1, thereby up-regulating the transcription factors required for the formation of neural precursor cells in normal brain tissue. The brain-specific Cdc42 isoform, Cdc42b, is essential for promoting the transformation of neural progenitor cells into neurons. Cdc42b and activated ACK jointly down-regulated mTOR expression and promoted neuronal differentiation 145. Alzheimer's disease (AD) and Parkinson' s disease (PD) are the two most common neurodegenerative diseases 146. In normal organisms, Cdc42 requires activation of multiple GEFs (Dbs, intersectin, Prex1, Tiam1, and Vav1-3) to promote neurite formation, axon growth and branching, spinal column formation, and neuronal differentiation 20. The overexpression of intersectin alter the Cdc42 mediated endocytosis, leading to the increased expression of Cdc42 in the prefrontal cortex of AD patients 147. The Cdc42/PAK complex controls neurite growth and filopodia formation. Cdc42 can promote the activation of PAK and PAK stimulates LIMK and inhibits the depolymerization of actin filaments. The imbalance of PAK/LIMK axis is the key to the formation of AD 148. In AD, the elevation of neuronal isoprenoid levels induces prenylation of Cdc42, which can activate GSK-3β, resulting in phosphorylation of Tau 149. In PD, synaptic dysfunction may cause neurons death by segregation of Cdc42/Rac in Lewy bodies. Recent study has shown that topoisomerase IIβ downregulation may cause neurodegeneration through dysregulation of Rho-GTPases leading to PD-like pathology, which may be a potential direction in the prevention and treatment of PD in the future 150. In addition, Huntington's disease is a progressive neuropsychiatric disorder associated with motor dysfunction due to neurotoxicity caused by the aggregation of huntingtin (Htt) protein. Cdc42 and Rac effector proteins promote Htt aggregation and exacerbate the effects of the disease. Damage to Cdc42 may also affect motor neuron death in amyotrophic lateral sclerosis 151.

## Small molecule inhibitors of Cdc42

There are many small molecule inhibitors of Cdc42. However, there are few clinical trials about Cdc42 inhibitors and most studies of cdc42 inhibitors are performed *in vivo* and *in vitro* in pre-clinical settings. Commonly used small molecule inhibitors of Cdc42 are listed in Table-1.

### Azathioprine decreases Cdc42 activity via blocking Rho-GEF

Most of the current inhibitors have been developed interact with GEF and bind nucleotides to inhibit Cdc42. Azathioprine (AZA) 1 and AZA197 are GEF interaction inhibitors. AZA1 can effectively inhibit the activation of Cdc42 and Rac1, reduce the signal transduction of the PAK pathway, and inhibit tumor growth. The main mechanism of AZA197 anti-tumor effect is through the inhibition of cell proliferation and induction of apoptosis. AZA197 destroys the interaction between Cdc42 and GEF and specifically inhibits the activity of Cdc42. AZA197 inhibits the proliferation, migration, and invasion of cancer cells in colon cancer and prostate cancer 152. It can reduce the activity of PAK and ERK signaling pathways and weaken the expression of cyclin D1, thus reducing the invasion and growth of colon cancer tumors. AZA197 treatment results in the transformation of the actin cytoskeleton and cellular morphology and prevents filopodia formation in colon cancer cells. AZA197 can effectively modulate PAK/ERK signaling transduction and interfere with cyclin D1 expression, thus affecting colon cancer cell proliferation 153.

### Casin targets regulation of Cdc42

Casin controls GEF activity by blocking PIP2 on the Cdc42/RhoGDI complex and inhibiting Cdc42 nucleotide exchange and further targeted regulation of Cdc42 154. It disrupts intersectin, thereby inhibiting the activation of Cdc42. Study have shown that in the hematopoietic stem cell system, the application of Casin can transform aging hematopoietic stem cells into young hematopoietic stem cells due to the inhibition of Cdc42 function^155^. Casin is widely used in cell processing, and different concentrations play different roles 156.

### MBQ167 is a GEF interaction inhibitor

MBQ167 is an effective dual inhibitor of Rac and Cdc42. In *silico*, MBQ167 is shown to bind to the Asn 39 side chain of Cdc42 and Rac to form an H bond, which is thought to inactivate the GEF binding region 157. MBQ167 shows improved efficacy in metastatic breast cancer cells by inhibiting Rac activity and Cdc42 activity. MBQ167 rearranges the actin cytoskeleton and adhesion plaques, resulting in loss of cell polarity and attachment to the extracellular matrix, as well as in a significant reduction in Rac-mediated lamellipodia/invadopodia and Cdc42-induced microtubules and filamentous pseudopodia. MBQ167 can effectively inhibit the activity of overall PAK because it induces a decrease in the activating phosphorylation (Y507/T508) of the direct PAK substrate LIMK and the inactivating phosphorylation (S3) of cofilin (actin depolymerization factor, a downstream effector of LIMK) 158. In addition, a group of PAK small-molecule inhibitors (such as FRAX486, 597, and 1036) and PAK-specific inhibitors have a similar function with favorable clinical efficacy (such as G-5555, NOV3, and AZ137-05339) 157, 159, 160. FRAX486 reduced audiogenic seizures. FRAX597 inhibits the proliferation, invasion, and metastasis of pancreatic cancer cells and Schwannoma cells, and decreases the progression of pancreatic cancer and neurofibroma type 2 161, 162.

### ZCL inhibits the progress of cancer by preventing EGFR/kRas

ZCL compounds inhibit cell proliferation and cell cycle progression by inhibiting the EGFR-kRas signaling pathway. Study has shown that ZCL278 plays an important role in inhibiting the invasion and metastasis of pancreatic cancer cells 163. ZCL278 can form two hydrogen bonds with Thr35 and Asp57, and hydrophobic interactions with Val36 and Thr35 of Cdc42. ZCL278 has a markedly increased affinity with Cdc42 in its GEF-complex conformation, promoting the binding of GTP and Cdc42. Additionally, ZCL278 can interact with Cdc42 in the corresponding intersectin groove, thereby acting as an antagonist and agonist. ZCL278 can act as a partial agonist for Cdc42, whereas ZCL367 is a Cdc42 inhibitor. ZCL367 inhibited the metastasis of lung cancer cells A549 and prostate cancer cells PC3 in a time-dependent manner. Compared with ZCL278, ZCL193, ZCL251 ZCL254, ZCL257, ZCL269, ZCL367 can effectively restrain microspike/filopodia formation of 3T3 cells. ZCL367 can form three hydrogen bonds with Asp38, Asn39, and Asp57, and hydrophobic interactions with Phe56 and Val36 of Cdc42. Because ZCL367 forms more hydrogen bonds than ZCL278, it is able to interact more strongly with Cdc42. ZCL367 has good selectivity to Cdc42 *in vitro* and inhibits the interaction between Cdc42 and intersectin in lung cancer A549 cell lysates. ZCL278 is also a GEF interaction inhibitor. It can destroy the interaction of ITSN with Cdc42 and on the surface of the Cdc42 Phe56 wide-ranging combination, inhibiting migration in the PC-3 cell line without destroying cell vitality and suppressing cellular invasion and migration in pancreatic cancer cell lines 164.

### ML141 reduces Cdc42 activity through interdicting GTP binding domain

ML141, is a competitive inhibitor of Cdc42 activity. It is a type of nucleotide inhibitor and non-steroidal anti-inflammatory drug (NSAID)-related compound, which can specifically block the GTP binding domain 165. It can reduce filopodia formation in fibroblasts induced by bradykinin, and significantly inhibit Cdc42 activity 165. CID44216842 is a homologous analog of ML141. The two drugs can effectively inhibit the migration of ovarian cancer cells 166. ML141 can also inhibit metastasis of breast cancer cells 166. Other NSAIDs, such as R-Ketorolac and R-Naproxen, can reduce the adhesion, metastasis, and invasion of ovarian cancer cells by inhibiting Cdc42 activity 167, 168. Ketorolac, a kind of Cdc42 specific inhibitor, can potentially contribute to the observed survival benefit in women after ovarian cancer surgery. Ketorolac has a novel pharmacologic activity by inhibiting Rac1 and Cdc42, potentially contributes to the survival benefit in women with ovarian cancer and reduces ovarian cancer-specific mortality 169.

### Secramine alleviates Cdc42 effect by blocking Rho-GDI

Secramine inhibits actin assembly *in vitro*, which is stimulated by PIP2 and mediated by the Cdc42/Toca-1/N-WASP/Arp2/3 signaling pathway 170. Secramine belongs to the RhoGDI modulators and exerts its function strictly depending on the presence of RhoGDI1. RhoGDI1 can promote the solubility of prenylated Cdc42 and act as a shuttle for the transport of GDP-Cdc42 or GTP-Cdc42 between the cytosol and target membranes 171. Secramine isolates Cdc42 and blocks isoprene Cdc42 from the cell membrane. It inhibits protein transport from the Golgi body to the plasma membrane 172. Secramines A and B do not require nucleotide exchange for nonprenylated Cdc42 because they do not interfere directly with the GEF, and do not compete with nucleotide binding. As a Cdc42-GDP -RhoGDI1 complex, Secramines A and B decrease the membrane association of prenylated Cdc42 (GDP) that is present in PIP2 liposomes. Secramine A inhibits Cdc42 activation and reduces the possibility of Cdc42 binding to the membrane by dissolving the PIP2 liposome or binding to the PIP2 inositol head 173.

## Conclusion

In this review, we described the structure and functions of Cdc42 and its family, analyzing its major regulatory effectors, and summarizing the drugs that specifically target Cdc42 in disease treatment. Cdc42 is widely involved in the regulation of human malignant and non-neoplastic diseases, and plays an important role in the invasion and metastasis of tumors, cell proliferation, and cell polarity. Furthermore, the potential regulatory role of Cdc42 in non-tumor diseases and malignant diseases is discussed. So far, although some inhibitors of Cdc42 protein have been developed, few drugs can be used in clinical treatment, and the regulatory mechanisms of Cdc42 in a variety of diseases need to be further explored in order to achieve effective treatment.

## Figures and Tables

**Table 1:** Small molecule inhibitors of Cdc42.

Inhibitors	Function	References
AZA1	AZA1 can interact with GEF and bind nucleotides to inhibit the activation of Cdc42 and Rac1.	152, 153
AZA197	AZA197 inhibits cell proliferation and induces apoptosis.	
Casin	Casin blocks PIP2 on the Cdc42/RhoGDI complex and inhibits Cdc42 nucleotide exchange and disrupts intersectin.	154-156
MBQ167	MBQ167 can inhibit Rac and Cdc42.	158
FRAX486, 597, 1036	PAK small-molecule inhibitors	157, 159-162
G-5555, NOV3, AZ137-05339	PAK-specific inhibitors	
ZCL	ZCL inhibits the progression of cancer by preventing EGFR/kRas.	163, 164
ML141	ML141 reduces Cdc42 activity through interdicting GTP binding domain.	165-167, 169, 174
Secramine	Secramine alleviates Cdc42 effect by blocking Rho-GDI	170-173

## Abbreviations

Rac1: ras-related protein 1
Cdc42: cell division control protein 42
ACK1: activated Cdc42 kinase 1
IQGAPs: IQ motif-containing GTPase-activating proteins
GEF: guanine nucleotide exchange factor
GAPs: GTPase activating proteins
Rnd: Rho-related GTP-binding protein
NF-ƙB: nuclear factor ƙB
JNK: c-Jun NH2-terminal kinase
MAPK: mitogen-activated protein kinase
N-WASP: neural wiskott-aldrich syndrome protein
Arp2/3: actin-related protein 2/3
PAKs: p21-activated kinases
GDIs: GTPase dissociation inhibitors
PAR: proteinase-activated receptor
PI3K: phosphoinositide 3-kinases
ERK: extracellular signal-regulated kinase
Toca-1: Cdc42-dependent actin assembly 1 (Toca-1)
MAP: microtubule-associated proteins
MKK: mitogen-activated protein kinase kinases
EMT: epithelial-mesenchymal transformation
HIF: hypoxic inducible factor
IL: interleukin
FGF: fibroblast growth factors
EGFR: epidermal growth factor receptor
ROCK: Rho-associated protein kinase
Asef: APC-stimulated guanine nucleotide exchange factor
APC: adenomatous polyposis coli
SmurF1: smad ubiquitylation regulatory factor-1
GSK3: glycogen synthase kinase
ERK1/2: extracellular signal-regulated kinase 1/2
Slit-Robo1: Slit homolog protein/roundabout homolog 1
TWEAK: TNF-like weak inducer of apoptosis
Fn14: fibroblast growth factor inducible-14
srGAPs: Slit-Robo1 GTPase activating protein
STAT6: signal transducer and activator of transcription 6
GATA3: transcription factor GATA-binding protein 3
Abeta: amyloid-β peptides 1-40 and 1-42
LIMK: LIM kinase
AD: alzheimer desease
PD: parkinson's disease
NSAIDs: non-steroidal anti-inflammatory drugs
